# A Combined Intranasal and Parenteral Vaccination Strategy Reduces Morbidity and Antimicrobial Treatments Associated with Bovine Respiratory Disease in Danish Commercial Veal Calves

**DOI:** 10.3390/pathogens15080826

**Published:** 2026-08-06

**Authors:** Henrik Schmidt, Anne Mette Hostrup Kjeldsen, Jonna Hjorth, Henrik Læssøe Martin

**Affiliations:** 1 HIPRA NORDIC, Ådalen 7C, 6600 Vejen, Denmark; jonna.hjorth@hipra.com; 2 SEGES Innovation, Agro Food Park 15, 8200 Aarhus N, Denmark; amk@seges.dk (A.M.H.K.); hlm@seges.dk (H.L.M.)

**Keywords:** bovine respiratory disease, antimicrobial use, veal calves, intranasal vaccination, parenteral vaccination

## Abstract

Background: Bovine respiratory disease (BRD) is a major driver of antimicrobial use in veal calf production systems. Although vaccination may reduce disease incidence and antimicrobial treatments, field evidence supporting clinically relevant reductions in antimicrobial use remains limited. This study evaluated the clinical efficacy of a combined intranasal (IN) and parenteral (subcutaneous/intramuscular; SC/IM) vaccination strategy targeting bovine respiratory syncytial virus, *Histophilus somni*, and *Mannheimia haemolytica* under commercial conditions. Methods: A randomised controlled field trial was conducted on two Danish veal calf farms between November 2019 and April 2020, including 880 calves allocated to a vaccinated group (*n* = 445) or an unvaccinated control group (*n* = 435). Health outcomes included morbidity, antimicrobial use, mortality, growth performance, and slaughterhouse pulmonary and pleural lesions and were analysed using mixed-effects models. Results: Vaccination significantly reduced BRD-associated morbidity across all evaluation periods (from day 5 after arrival to slaughter), with relative reductions of 18.8–24.6% and 30% fewer antimicrobial treatment days. Pulmonary and pleural lesions were significantly less frequent in vaccinated calves (≈36% reduction). No statistically significant differences were observed in growth performance or mortality. Conclusions: Combined IN + SC/IM vaccination was associated with lower BRD-associated morbidity, antimicrobial treatments, and slaughterhouse respiratory lesions under commercial conditions.

## 1. Introduction

Bovine respiratory disease (BRD) is the most common and severe health condition affecting calf-rearing herds [1]. The disease represents a persistent challenge during the early post-arrival period [2], when calves are exposed to multiple risk factors, including calf-related characteristics, transportation, mixing of different origins, along with other changes in environmental and management practices [3,4]. Beyond its immediate clinical impact, BRD has substantial short- and long-term consequences for animal health and productivity. Affected calves experience increased morbidity and mortality and reduced growth, which together compromise lifetime performance [2,5,6]. Subclinical respiratory disease has even been associated with impaired growth and long-lasting effects on productive efficiency [7], underscoring the importance of effective early-life prevention.

BRD is a multifactorial disease resulting from interactions between infectious agents, host immune response, and management-related stressors [8]. Viral pathogens such as bovine respiratory syncytial virus (BRSV), together with bacterial agents including *Mannheimia haemolytica* (MH) and *Histophilus somni* (HS), play a central role in disease initiation and progression [1,8,9]. The clinical management and control of these pathogens rely heavily on antimicrobial therapy, making BRD one of the leading contributors to overall antimicrobial consumption in the cattle sector, with a substantial economic burden arising from treatment costs and associated production losses [10,11,12].

Consequently, public concern and regulatory pressure have increased to mitigate antimicrobial resistance (AMR) associated with extensive antimicrobial use in livestock production. European policies emphasise the need to decrease antimicrobial consumption together with the implementation of preventive measures as key strategies in protecting animal and human health within a One Health framework [13,14]. For instance, vaccination programmes may contribute to a reduction in antimicrobial use while preserving performance indicators in beef production systems [15].

In Denmark, respiratory disease accounts for the majority of antimicrobial consumption in veal calf production, particularly in systems characterised by intensive calf purchase and commingling from multiple suppliers [16,17]. National surveillance reports further show that antimicrobial use is concentrated in young cattle (<12 months of age), reinforcing the importance of preventive strategies during early production stages [18]. In response, national plans have been implemented to promote prudent antimicrobial use in food animals and mitigate AMR within the One Health strategy [19], supported by integrated surveillance systems that monitor antimicrobial consumption and resistance patterns (e.g., DANMAP; [20]).

Danish beef production relies on pure-breed dairy bull calves and an increasing number of crossbred beef-from-dairy calves (bull and heifer calves), which are transferred to specialised veal calf producers at approximately 14 days to two months of age, a period associated with increased susceptibility to BRD. Although vaccines against respiratory pathogens have become more widely available, their adoption has historically been influenced by heterogeneous field evidence, with reported outcomes varying according to the production system and age at vaccination [21,22]. One of the main challenges in vaccinating young calves is the presence of maternally derived antibodies (MDAs), which can interfere with immune responses induced by parenterally administered vaccines [23,24]. In contrast, intranasal (IN) vaccines have been shown to bypass MDAs and stimulate local mucosal immunity in the upper respiratory tract under experimental conditions, providing earlier protection against BRD pathogens [23,25]. Controlled field trials conducted in commercial calf-rearing units have reported encouraging effects of IN vaccination on BRD-associated outcomes; however, the reported effects on mortality and growth performance have been inconsistent across field studies [26,27,28]. Additionally, evidence regarding the impact of IN vaccination on decreasing antimicrobial use under field conditions remains limited, with reported reductions considered of limited clinical relevance [29].

Therefore, the main objective of this controlled field study was to evaluate the clinical efficacy of a combined IN and subcutaneous/intramuscular (SC/IM) vaccination strategy targeting BRSV, HS and MH in veal calf production. Particularly, this study aimed to assess the impact of vaccination on BRD-associated morbidity and antimicrobial use under commercial field conditions. Secondary objectives included the evaluation of mortality, growth performance, and slaughterhouse pulmonary and pleural lesions.

## 2. Materials and Methods

### 2.1. Animals, Study Population and Housing

This study was conducted between November 2019 and April 2020 on two commercial Danish veal calf farms (Farm A and Farm B) with known problems of respiratory disease occurring after calf arrival. A total of 880 calves originating from multiple Danish dairy farms and belonging to nine different consecutive batches were enrolled upon arrival at the fattening units.

Upon arrival, calves were approximately one month of age, with a mean age of 33.9 days in the vaccinated group and 36.3 days in the control group. Mean body weight at entry was 63.8 kg in vaccinated calves and 67.8 kg in control calves. The detailed distribution of age and weight by farm and treatment group is provided in Table 1. Overall, 445 calves were assigned to the vaccinated group and 435 calves to the control group. On farm A, 321 male calves were enrolled, including 318 large-breed calves and 3 beef crossbreeds. On farm B, the population consisted predominantly of male calves (388 large-breed calves and 89 beef crossbreeds), while a limited number of heifers were included, comprising 1 large-breed calf and 81 beef crossbreeds, reflecting normal commercial practice. None of the animals had received any previous vaccinations prior to enrolment in the study.

During the initial study period, calves were housed in adjacent pens under similar environmental conditions but without physical contact between pens, ensuring biosecurity separation between vaccinated and control calves. The initial period extended up to day 128 after arrival at Farm A and day 86 after arrival at Farm B, reflecting differences in standard farm management.

After completion of the initial period, calves were regrouped and integrated into the farms’ standard production flow, during which vaccinated and control animals could be mixed for the remainder of the study period until slaughter, in accordance with normal commercial management.

Stocking density and pen size followed standard commercial recommendations for Danish slaughter calf production. On Farm A, calves were housed at approximately 2.3 m^2^ per calf during the first 7 weeks after arrival, increasing to 3.5 m^2^ per calf for the rest of the study period. On Farm B, stocking density was approximately 3.0 m^2^ per calf throughout the study period.

Feeding and general management followed the standard routines of each farm. Calves received milk replacer during the early rearing phase, with a gradual transition to solid feed. Starter feed was available *ad libitum*, and calves had continuous access to drinking water throughout the study period. No experimental modifications to feeding or management practices were introduced.

Housing facilities were typical for Danish veal calf systems. Pens were bedded with straw, which was replenished according to routine farm management, and buildings relied on natural ventilation; however, specific parameters such as air exchange rates were not recorded. Environmental temperature and humidity were not monitored as part of the study.

### 2.2. Baseline BRD Status

At the beginning of the study, tracheal lavage samples were collected from a subset of calves on one of the participating farms (Farm B, *n* = 3) and analysed by PCR to characterise the baseline respiratory pathogen profile. PCR analysis confirmed the presence of MH, *Mycoplasma bovis* (MB), *Pasteurella multocida*, and HS (one sample), as well as respiratory viruses including BRSV (two samples) and bovine coronavirus (one sample).

Other viral pathogens relevant to BRD, including infectious bovine rhinotracheitis virus and bovine viral diarrhoea virus, were unlikely to have contributed to respiratory disease on the participating farms, as Denmark has a free status and both diseases are subject to national surveillance and control programmes [30].

### 2.3. Study Design

This study was designed as a randomised, controlled field trial under commercial conditions. Randomization was performed at the pen level within matched pairs of pens, consisting of approximately 10 calves per pen on Farm A and 12 calves per pen on Farm B. The matched pairs of pens were balanced for age, body weight, breed type, and sex. Pens within each matched pair were assigned to either the vaccinated group or the non-vaccinated control group. There was no physical contact between groups during the initial period.

Health monitoring and treatment decisions were performed by farm personnel blinded to vaccination status.

### 2.4. Vaccination Protocol

Calves assigned to the vaccinated group received a combined vaccination program targeting viral and bacterial respiratory pathogens, while control animals remained unvaccinated. The vaccination scheme consisted of the following administrations:

1. Intranasal vaccination with NASYM^®^ (1 mL in each nostril; Laboratorios Hipra S.A., Amer, Spain), a modified-live vaccine containing BRSV, administered upon arrival at the veal calf farm (day 0).
2. Intramuscular booster vaccination with NASYM^®^ (2 mL), approximately 7 weeks after arrival (day 49).
3. Subcutaneous vaccination with HIPRABOVIS^®^ SOMNI/Lkt (2 mL; Laboratorios Hipra S.A., Amer, Spain), an inactivated vaccine targeting HS and MH leukotoxoid, administered at the time of the intramuscular NASYM^®^ booster (day 49).
4. A second subcutaneous dose of HIPRABOVIS^®^ SOMNI/Lkt (2 mL), administered 3 weeks later (day 70), according to the manufacturer’s recommendations.

Vaccinations were performed by the investigator or by veterinarians trained and instructed for the study. There were no farm personnel present during the vaccination procedures. Vaccines were stored, handled, and administered in accordance with the manufacturers’ instructions.

### 2.5. Disease Monitoring and Treatment Criteria

Calves were monitored for health status throughout the study period by farm personnel trained by veterinarians involved in the project. Clinical inspections were performed daily in accordance with normal farm routines.

Disease detection was based on the presence of clinical signs compatible with respiratory disease, including cough, nasal and/or ocular discharge, abnormal ear position, and elevated rectal temperature according to the clinical scoring system described by McGuirk [31]. In addition, clinical signs including depression or reduced alertness and increased respiratory rate or laboured breathing were incorporated into the clinical assessment used to identify calves with respiratory disease. Calves presenting clinical signs compatible with disease received therapeutic intervention for respiratory disease based on the clinical judgement of trained personnel.

Treatments were administered according to each farm’s established treatment protocols and in consultation with the herd veterinarian when required. It should be emphasised that no prophylactic or metaphylactic treatment was used during the study period.

### 2.6. Outcomes

#### 2.6.1. Health and Clinical Data

Mortality, morbidity (defined as the proportion of calves treated for respiratory disease), and detailed individual treatment records, including product, dosage, and treatment duration, were retrieved from the national Danish Cattle Database (DCDB, owned by the Danish Agriculture & Food Council and managed by SEGES Innovation P/S, Aarhus N, Denmark).

Treatment duration was defined as the number of effective antimicrobial treatment days (daily treatment doses), calculated based on the expected duration of action of each antimicrobial product as described in the Danish Veterinary and Food Administration’s veterinary register database VetStat (Table 2), relative to the number of days the calf was included in the initial period (days 5–86 after arrival).

Only antimicrobial products were included in the calculation of treatment duration. Non-antimicrobial products, including non-steroidal anti-inflammatory drugs were excluded from the analysis.

Day 5 after arrival was defined as the start of the study period to reflect the expected early onset of protection following intranasal vaccination, based on the reduction in respiratory clinical signs from day 5 after vaccination reported in the product characteristics [32]. Health outcomes were analysed for the period’s days 5–49 (~2nd vaccination), days 5–86 (regrouping), and days 5 until slaughter (overall study period).

#### 2.6.2. Performance Data

Calves were weighed upon arrival at the veal calf farm, approximately 7 weeks post-arrival (day 49), at regrouping (between 86 and 128 days post-arrival, depending on farm), and at slaughter.

Growth performance was evaluated by calculating average daily gain (ADG) over predefined study periods. Routine slaughterhouse data, including slaughter remarks (lung and pleural lesions compatible with respiratory disease), were retrieved from the DCDB.

### 2.7. Statistical Analysis

The study was originally designed by simulations to include 476 calves per group to detect a reduction in the proportion of calves treated for respiratory disease from 80 to 60%, assuming a significance level of α = 0.05 and 80% statistical power. With this sample size, a difference in ADG of at least 50 g/day was expected to be detectable. The final number of enrolled calves was 445 in the vaccinated group and 435 in the control group.

The individual calf was considered the experimental statistical unit. Missing data were handled by excluding incomplete observations from the respective analyses without imputation. Differences were considered statistically significant at *p* < 0.05. Statistical analyses were performed using SAS software (Statistical Analysis System, version 9.4, SAS Institute Inc., Cary, NC, USA).

Baseline descriptive variables are presented as means and standard deviations. Model-based outcomes are reported as least squares means (LSM) with corresponding non-adjusted 95% confidence intervals (CI), as appropriate. Binary outcomes (e.g., treatment for respiratory disease, slaughter remarks, mortality) were analysed using generalised linear mixed models with a logit link function (PROC GLIMMIX), whereas continuous outcomes (e.g., ADG, age at slaughter, and treatment duration) were analysed using linear mixed models (PROC MIXED). Treatment duration was log-transformed using the function log (daily doses/days in risk period +0.05). The offset was added to allow inclusion of calves with no antimicrobial treatment days. As the proportion of treatment days was generally lower in the vaccinated group, this offset reduces the relative contrast between the vaccinated and control calves. Therefore, any significant treatment effect detected after transformation is unlikely to be caused by artificial inflation of differences among calves with a few treatment days.

Treatment group and the interaction between treatment group and herd were included in the models as fixed effects, while batch and pen (nested within batch) were included as random effects to account for clustering. Covariates—including herd, sex, breed type, and the quadratic polynomial of entry body weight—were included when relevant.

## 3. Results

### 3.1. Morbidity and Treatments

**Figure 1:**
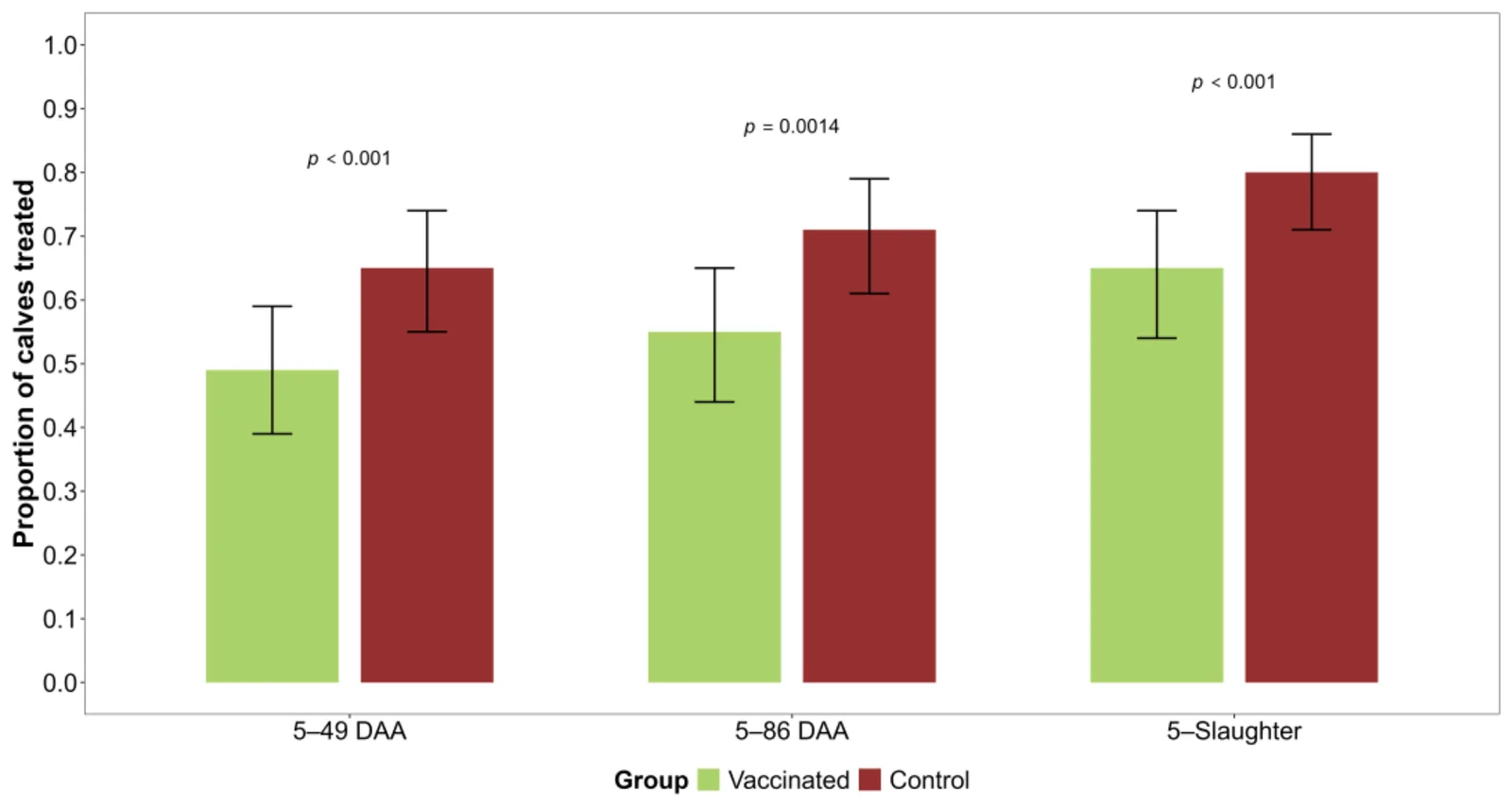
Estimated proportion of calves treated for lung diseases from day 5 after arrival to different time intervals. Error bars indicate 95% confidence intervals. DAA, days after arrival.

The estimated proportion of calves treated for lung disorders was consistently lower in the vaccinated group compared with the control group across all evaluated time intervals (Figure 1). Between days 5 and 49 after arrival, vaccinated calves exhibited a significantly lower estimated proportion of animals treated for lung disorders (0.49; 95% CI: 0.39–0.59) compared with control calves (0.65; 95% CI: 0.55–0.74; *p* < 0.001), corresponding to a 24.6% relative reduction in treatment incidence during the early post-arrival period.

A similar significant reduction was observed for the period from days 5 to 86 after arrival, with estimated proportions of 0.55 (95% CI: 0.44–0.65) in vaccinated calves and 0.71 (95% CI: 0.61–0.79) in control calves (*p* = 0.0014), representing a relative reduction of 22.5% in the vaccinated group compared with controls. From day 5 after arrival until slaughter, the estimated proportion of calves treated for lung disorders remained significantly lower in the vaccinated group (0.65; 95% CI: 0.54–0.74) compared with the control group (0.80; 95% CI: 0.71–0.86; *p* < 0.001), corresponding to a relative reduction of 18.8% over the overall study period.

**Figure 2:**
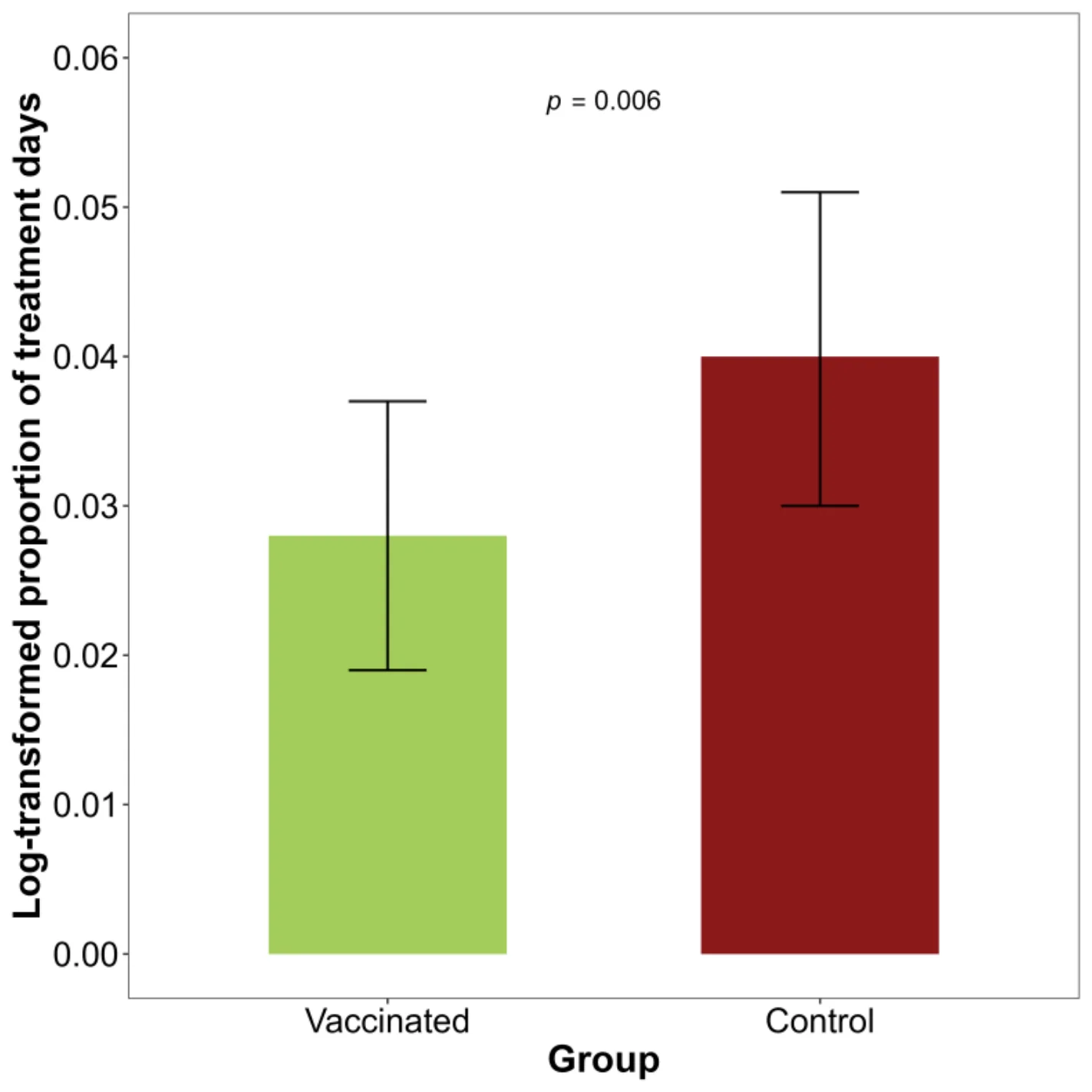
Estimated least squares mean of the log-transformed proportion of treatment days during days 5–86 after arrival. Error bars indicate 95% confidence intervals.

The estimated least squares mean (LSM) of the log-transformed proportion of treatment days for respiratory tract infections was significantly lower in vaccinated calves than in control calves (Figure 2). During days 5–86 post-arrival, vaccinated calves had a lower mean log-transformed proportion of treatment days (LSM = 0.028; 95% CI: 0.019–0.037) compared with control calves (LSM = 0.040; 95% CI: 0.030–0.051), representing a statistically significant difference between groups (*p* = 0.006) and an approximate 30% relative reduction in treatment days for respiratory disease in the vaccinated group.

### 3.2. Slaughter Remarks

**Figure 3:**
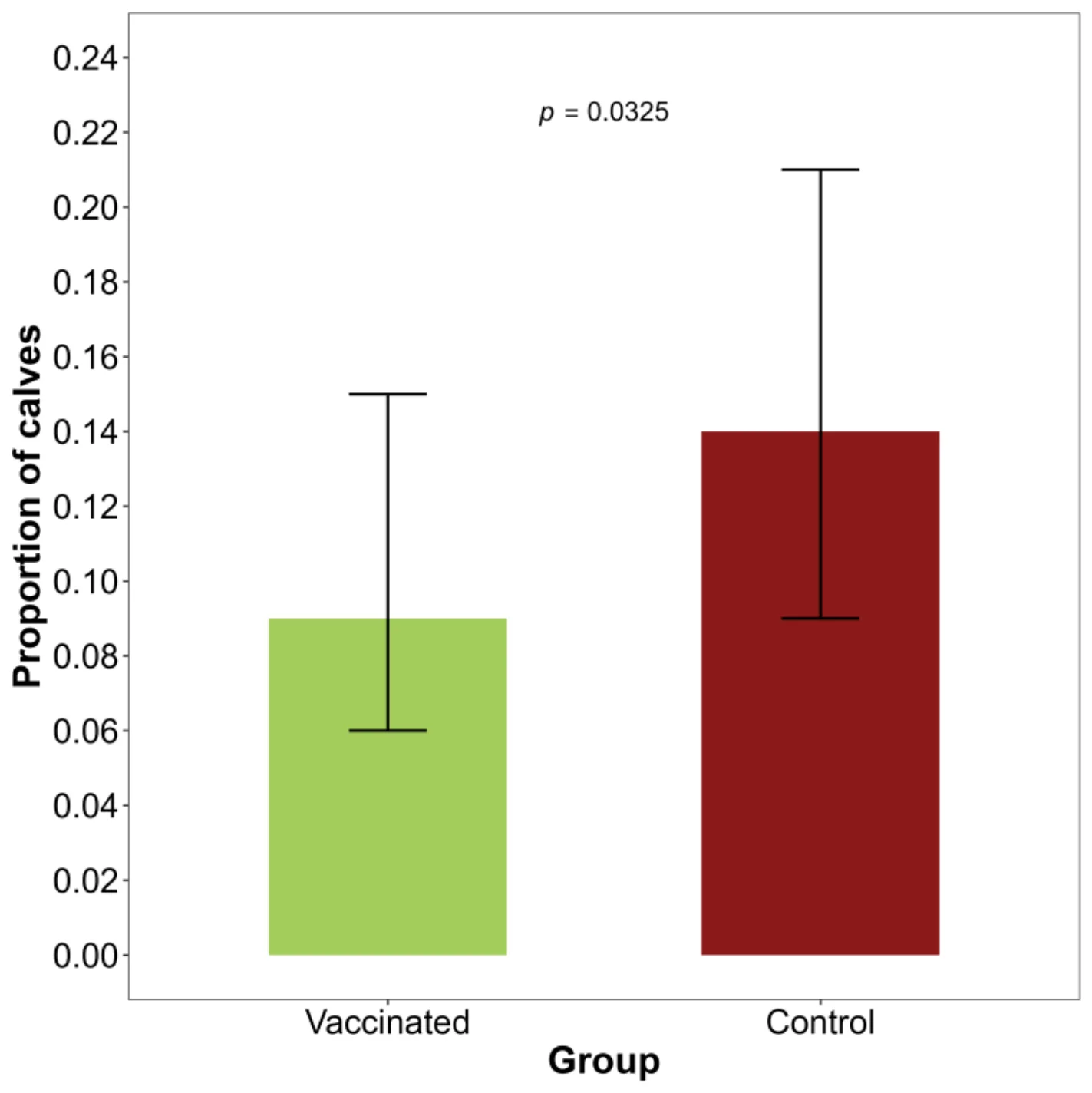
Estimated proportion of calves with remarks for pulmonary and pleural disorders from slaughterhouses. Error bars indicate 95% confidence intervals.

The proportion of calves presenting remarks related to lung or pleural disorders was significantly lower at slaughter in the vaccinated group compared with the control group (Figure 3).

The estimated proportion of affected calves was 0.09 (95% CI: 0.06–0.15) in the vaccinated group and 0.14 (95% CI: 0.09–0.21) in the control group, with a statistically significant difference between groups (*p* = 0.0325), corresponding to a relative reduction of approximately 35.7% in the occurrence of lung and pleural lesions in vaccinated calves.

### 3.3. Performance and Mortality

LSM estimates for growth and age-at-slaughter parameters, as well as estimated proportions for mortality outcomes, are summarised in Table 3. Growth during the first seven weeks of life did not differ significantly between vaccinated calves (LSM = 840.1 g/day) and control calves (LSM = 816.3 g/day; *p* = 0.133), although values were numerically higher in the vaccinated group. Likewise, no significant difference was observed in age at slaughter, with LSM values of 326.2 days in the vaccinated group and 327.0 days in the control group (*p* = 0.720). Net growth at slaughter was also comparable between groups, with LSMs of 616.5 g/day for vaccinated calves and 608.2 g/day for control calves (*p* = 0.155), again showing numerically higher values in vaccinated animals.

Mortality outcomes, expressed as estimated proportions, did not differ significantly between vaccinated and control calves at any evaluated time point. The estimated proportion of deaths at 49 days was 0.006 in vaccinated calves compared with 0.012 in control calves (*p* = 0.291), corresponding to a 50% relative reduction. At 86 days, the corresponding proportions were 0.011 and 0.023, respectively (*p* = 0.143). Over the entire study period, the estimated proportion of deaths was 0.019 in the vaccinated group and 0.031 in the control group (*p* = 0.165).

## 4. Discussion

The present field study conducted under commercial veal calf production conditions demonstrated significant reductions in BRD-associated morbidity and antimicrobial treatments across all evaluated time intervals following a combined IN and parenteral SC/IM vaccination strategy. Additionally, the protocol was associated with a significant reduction in slaughterhouse pulmonary and pleural lesions. In contrast, growth performance parameters and mortality did not differ significantly between groups. Overall, the results suggest that the vaccination strategy may contribute to reducing BRD-associated morbidity and antimicrobial treatment use without negatively affecting production parameters, thereby supporting current national antimicrobial reduction strategies.

Previous field studies of IN BRD vaccination in young calves have reported variable results. For example, Sandelin et al. [27] did not observe a significant vaccination effect on morbidity at first clinical diagnosis, antimicrobial use, or mortality in a commercial Finnish calf-rearing unit, despite relatively high BRD incidence (>35%) and BRD treatment rates (>30%), slightly lower than those observed in this study. Mustonen et al. [29] reported that more than 90% of calves were treated for respiratory infection in a Finnish calf-rearing unit and observed a significant reduction in antimicrobial use following vaccination, although the clinical relevance was considered modest. Growth performance was not affected, while mortality was reduced in vaccinated calves. Similarly, Ollivett et al. [26] reported reductions in ultrasonographic lung consolidation but inconsistent effects on clinical disease and growth across Holstein dairy calf herds. Other studies reported no differences in morbidity or antimicrobial treatment between protocols comparing IN and parenteral vaccination [28]. More recently, Jourquin et al. [33] showed a significant reduction in ultrasonography-confirmed pneumonia in male dairy calves after on-arrival IN–parenteral vaccination. However, individual antimicrobial treatments were not evaluated because calves received metaphylactic therapy during the outbreak period. In contrast, the present study found statistically significant reductions in morbidity (approximately 20–25%), antimicrobial use (near 30%), and slaughterhouse lesions (36%) associated with a combined IN and parenteral SC/IM vaccination strategy. Although these relative reductions corresponded to more modest absolute differences, particularly for treatment days and slaughterhouse lesions, they may still be relevant in high-risk veal calf systems where BRD is a major driver of antimicrobial use. The results suggest that the protocol may contribute to the reduction in antimicrobial use and, potentially, the reduction in the selection pressure for AMR, although AMR risk outcomes were not directly assessed. The reduction in slaughterhouse pulmonary and pleural lesions may also be economically relevant, as BRD and lung lesions have been associated with impaired carcass weight and quality and poorer economic outcomes [7,34]. In addition, reduced morbidity and treatment requirements may also decrease workload on farms, as a substantial proportion of labour time and costs associated with BRD management is related to animal handling and treatment administration [35].

Regarding the vaccination strategy, the combination of early mucosal priming and a subsequent systemic booster may provide a broader time window of protection than single-route strategies. In this study, IN administration of a modified-live BRSV vaccine on arrival was followed by an IM booster at day 49, supplemented by two SC doses of an inactivated vaccine targeting bacterial HS and MH leukotoxoid at days 49 and 70. This approach was designed to leverage the complementary mechanisms of IN and parenteral vaccination routes. IN priming stimulates rapid local mucosal immunity, including secretory IgA and cell-mediated responses, providing early protection during the first week after vaccine administration [25,36]. Importantly, this early mucosal activation occurs independently of MDA levels, thereby circumventing the MDA-mediated suppression of immune responses that typically limits parenteral vaccination in young calves [23,24]. Indeed, Marzo et al. [25] demonstrated that the vaccine used in the present study conferred protection against experimental BRSV challenge in calves with high MDA titres, confirming the ability of IN vaccination to bypass this interference. Additionally, heterologous parenteral boosting amplifies systemic neutralising antibody responses [37] and generates immune responses comparable to homologous parenteral protocols, with the added advantage of earlier mucosal priming [38]. The multi-dose approach used in the present study contrasts with the single-dose IN protocols used by Sandelin et al. [27], which may partially explain the larger and more consistent reductions in antimicrobial treatment observed across all evaluation periods with the heterologous multi-dose, multi-route vaccination strategy.

The multifactorial nature of BRD and the complexity and unpredictability of commonly observed viral–bacterial interactions contribute to the complexity of this disease [1]. In young calves, primary viral infections are frequent, particularly those caused by BRSV [21,39,40,41]. These viral infections can predispose the respiratory tract to secondary bacterial colonisation and proliferation by other pathogens involved in BRD [9,15]. Among these, MH plays a key role in the development of bacterial pneumonia [42]. Increasing AMR has also been reported among BRD-associated bacterial pathogens, including reduced susceptibility to macrolides and tetracyclines [43]. In production systems characterised by high infection pressure and intensive antimicrobial use, such as Danish veal calf systems [16,17], frequent antimicrobial treatments may contribute to the selection of resistant bacterial populations. The exploratory baseline assessment detected several common BRD-associated pathogens on one of the farms in the present study, further supporting the relevance of vaccination strategies as preventive tools in reducing antimicrobial use, in line with One Health objectives. Under these conditions, vaccination strategies considering systemic boosters targeting bacterial components may provide broader protection than those targeting viral pathogens alone. In the present study, the combined IN and SC/IM vaccination protocol targeting both viral and bacterial components was associated with significant reductions in morbidity and antimicrobial treatments, as well as a marked decrease in slaughterhouse pulmonary and pleural lesions. These findings support the hypothesis that a vaccination strategy targeting both the viral priming phase and the bacterial components of the BRD complex may contribute to reducing BRD-associated morbidity and treatment requirements under commercial conditions, with extended benefits that could be detected even at the end of the cycle (at slaughter).

Furthermore, the relative contribution of individual pathogens varies across herds, regions, age groups and production systems. For instance, the prevalence of MH has been reported to be higher in feedlot or veal calves than in adult cattle [44]. In addition to pathogen-specific differences, management and environmental factors—including housing conditions, hygiene practices, colostrum management, commingling, or group size—may influence the occurrence and severity of respiratory disease in calves [21,45,46]. These factors can introduce variability in infection pressure between farms or groups and potentially confound the evaluation of preventive strategies under field conditions. In the present study, the two participating farms differed in the duration of the initial study period (128 vs. 86 days), stocking densities, and breed composition. Additional unmeasured factors, such as colostrum quality, environmental temperature and humidity, and transport-related stressors, may also have influenced the outcomes observed, although transport times in the present study were relatively short (generally 1–3 h). Taken together, these observations support the need for vaccination strategies tailored to herd structure, management conditions, and regional epidemiological risk [21]. However, the practical implementation of the protocol should also be considered, as the four-dose vaccination schedule over 70 days may limit adoption in commercial systems with high labour constraints or limited opportunities for repeated animal handling. Such risk-adapted approaches align with One Health principles, particularly in the context of reducing antimicrobial use and mitigating AMR [47], by supporting targeted vaccination strategies adapted to specific animal populations, production systems, and regional disease patterns as essential tools for effective disease control [48].

Despite the clear reduction in morbidity and antimicrobial treatments, vaccination did not result in statistically significant differences in growth performance or mortality between vaccinated and control calves. The study was powered to detect a difference of at least 50 g/day in ADG; therefore, smaller but potentially meaningful effects may not have reached statistical significance. Comparable findings have been reported in other field studies evaluating BRD vaccination, where improvements in growth or survival were not consistently significant [26,27,29], suggesting that larger sample sizes or multi-centre study designs may be needed to capture these outcomes. Additionally, several limitations should be considered when interpreting the present findings. The study was conducted on only two commercial veal calf farms with known BRD problems; therefore, the results may not be directly generalised to farms with lower disease pressure or different management systems. The baseline microbiological assessment was limited to three calves from Farm B and was intended only to provide contextual information on circulating BRD-associated pathogens, not to characterise pathogen dynamics across both farms, all batches, or all clinical cases. Although environmental variables such as temperature, humidity, and ventilation rates were not continuously monitored, vaccinated and control calves within each farm were housed contemporaneously under the same environmental conditions. Therefore, environmental variation is unlikely to have biassed comparisons between treatment groups, although its influence on the overall occurrence of BRD cannot be excluded. No immunological measurements or systematic PCR testing of clinical cases before antimicrobial treatment were performed; therefore, the mechanisms underlying the observed clinical effects and the pathogens responsible for individual BRD cases could not be confirmed. Finally, after the initial separated housing period, vaccinated and control calves could be mixed within the standard production flow, which may have attenuated differences between groups during the later study period.

## 5. Conclusions

This field-controlled study showed that a combined intranasal and parenteral vaccination strategy targeting BRSV, HS, and MH was associated with significant reductions in BRD-associated morbidity and antimicrobial treatments in Danish commercial veal calf production. The protocol was also associated with fewer slaughterhouse pulmonary and pleural lesions, suggesting improved respiratory health throughout the production cycle. Further studies including systematic microbiological, immunological, and economic assessments would help to better characterise the mechanisms and practical value of this vaccination strategy.

## Figures and Tables

**Table 1:** Description of study populations.

Farm (Batches)	Group	N Calves (Heifer Proportion)	Age at Entry (Days; Mean ± SD)	Input Weight (kg; Mean ± SD)
A (6)	Vaccinated	163 (0.0%)	44.3 ± 12.8	69.3 ± 9.8
	Control	158 (0.0%)	49.3 ±13.2	77.3 ± 11.1
B (3)	Vaccinated	282 (12.1%)	28.0 ± 12.0	60.7 ± 10.7
	Control	277 (17.3%)	28.9 ± 10.4	62.4 ± 11.1
Overall (9)	Vaccinated	445 (7.6%)	33.9± 14.6	63.8 ± 11.2
	Control	435 (11.0%)	36.3 ± 15.1	67.8 ± 13.2

SD, standard deviation. Upon arrival, all calves were checked for general health status by a veterinarian or trained personnel, and calves showing clinical signs requiring treatment upon entry were excluded from the study.

**Table 2:** Duration of action of antimicrobial products included in the calculation of treatment days (VetStat).

Number of Days with Effect ^1^	Product	Active Principle	Therapeutic Group
1 day	AQUACYCLINE Vet. (Ceva Santé Animale, Libourne, France)	Oxytetracycline	Tetracyclines
	ENGEMYCIN Vet. (Intervet International B.V., Boxmeer, The Netherlands)	Oxytetracycline	Tetracyclines
	ETHACILIN Vet. (Intervet International B.V., Boxmeer, The Netherlands)	Benzylpenicillin (penicillin G)	Penicillins (β-lactamase sensitive)
	NORODINE Vet. (Norbrook Laboratories, Newry, UK)	Trimethoprim + Sulfadiazine	Sulfonamides with trimethoprim
	TYLAN Vet. (Elanco Animal Health, Greenfield, IN, USA)	Tylosin	Macrolides
	BORGAL Vet. (Ceva Santé Animale, Libourne, France)	Sulfadoxine + Trimethoprim	Sulfonamides with trimethoprim
2 days	FLORKEM (Ceva Santé Animale, Libourne, France)	Florfenicol	Amphenicols
	ALAMYCIN PROLONGATUM Vet. (Norbrook Laboratories, Newry, UK)	Oxytetracycline	Tetracyclines
	RESFLOR Vet. (Intervet International B.V., Boxmeer, The Netherlands)	Florfenicol + Flunixin	Amphenicols + NSAID
	VETRIMOXIN L.A. (Ceva Santé Animale, Libourne, France)	Amoxicillin	Penicillins (extended spectrum)
5 days	ZUPREVO (Intervet International B.V., Boxmeer, The Netherlands)	Tildipirosin	Macrolides
	DRAXXIN (Zoetis, Parsippany, NJ, USA)	Tulathromycin	Macrolides

^1^ Source: https://vetstat.fvst.dk/vetstat/ (accessed on 1 May 2020); NSAID: non-steroidal anti-inflammatory drug.

**Table 3:** Least squares means (LSMs) for performance parameters and estimated mortality proportions.

Parameter	N	Control LSMs [95% IC]	Vaccinated LSMs [95% IC]	*p*-Value
ADG during first 7 weeks (g/day)	847	816.3 [778.5:854.1]	840.1 [801.8:878.4]	0.1331
Age at slaughter (days)	817	327.0 [322.1:332.0]	326.2 [321.2:331.3]	0.7204
ADG at slaughter (g/day)	817	608.2 [592.9:623.4]	616.5 [601.1:631.9]	0.1550
Proportion of deaths at 49 days	880	0.012 [0.004:0.032]	0.006 [0.002:0.019]	0.2909
Proportion of deaths at 86 days	880	0.023 [0.011:0.046]	0.011 [0.005:0.027]	0.1425
Proportion of deaths (overall)	880	0.031 [0.013:0.074]	0.019 [0.007:0.050]	0.1652

ADG, average daily gain.

## Data Availability

The data used in this study were obtained from the Danish Cattle Database (DCD) under a data-sharing agreement with the participating farms and are not publicly available. Restrictions apply to the availability of these data, as ownership remains with the farmers and sharing with third parties is not permitted. Information regarding the data processing and coding procedures may be obtained from A.M.H.K.

## Abbreviations

The following abbreviations are used in this manuscript:

ADG	Average Daily Gain
AMR	Antimicrobial Resistance
BRD	Bovine Respiratory Disease
BRSV	Bovine Respiratory Syncytial Virus
CI	Confidence Interval
DAA	Days After Arrival
DCDB	Danish Cattle Database
HS	*Histophilus somni* (HS)
IM	Intramuscular
IN	Intranasal
LSM	Least Squares mean
MB	*Mycoplasma bovis*
MDAs	Maternally Derived Antibodies
MH	*Mannheimia haemolytica* (MH)
NSAID	Non-Steroidal Anti-Inflammatory Drug
SC	Subcutaneous
